# Risk-adapted approaches to the management of clinical trials: guidance from the Department of Health (DH)/Medical Research Council(MRC)/Medicines and Healthcare Products Regulatory Agency (MHRA) Clinical Trials Working Group

**DOI:** 10.1186/1745-6215-12-S1-A39

**Published:** 2011-12-13

**Authors:** Sarah Meredith, Martyn Ward, Gillian Booth, Andrew Fisher, Carrol Gamble, Heather House, Martin Landray

**Affiliations:** 1 MRC Clinical Trials Unit, London, UK; 2 Clinical Trials Unit, Medicines and Healthcare Products Regulatory Agency, UK; 3 Clinical Trials Research Unit, Leeds University, Leeds, UK; 4 GCP Inspectorate, Medicines and Healthcare Products Regulatory Agency, UK; 5 MCRN Clinical Trials Unit, Liverpool University, Liverpool, UK; 6 Clinical Trials and Research Governance, Oxford University & John Radcliffe Hospital, Oxford, UK; 7 Clinical Trial Service Unit, Oxford University, Oxford, UK

## 

In 2009, the Department of Health asked the MRC and MHRA to identify major obstacles to non-commercial clinical trials research in the UK and suggest remedial actions. Risk-proportionality in trial management and monitoring was identified as a key area and a sub-group formed to:

• Develop a process to facilitate the agreement of key stakeholders on the level of risk associated with a clinical trial of an investigational medicinal product (CTIMP).

• Identify how risk-adapted approaches for CTIMPs can be achieved within the current regulatory framework.

• Develop guidance on risk assessment and the risk-proportionate management of clinical trials.

The resulting guidance focuses on the risks inherent in a trial protocol which impact on participant safety and rights, and the reliability of the results. A two-part assessment is suggested: 1) a simple IMP risk categorisation based on marketing status and standard medical care, and 2) assessment of the trial design, population and procedures to identify specific areas of vulnerability.

The first part, IMP risk category, has implications for simplifications of initiation and conduct of a CTIMP that may be possible within the current regulatory framework. Possible risk-adaptations include: the need for competent authority authorisation; content of the Clinical Trials Authorisation (CTA) application; IMP management; safety surveillance; trial documentation; and GCP Inspection. The risks associated with the IMP also determine trial procedures for monitoring participant safety.

The second part of the risk assessment addresses other aspects of clinical trial design and methods: safety risks from clinical procedures; risks related to participant rights; and risks to the reliability of trial results. It is designed to help trialists identify potential vulnerabilities and to prepare tailored trial management and monitoring plans to minimise risks which may be reviewed and modified throughout the life of a trial.

The IMP risk category and safety monitoring plan may be submitted to the MHRA with the CTA application to ensure that there is shared understanding on this key aspect of a trial. We hope that the entire risk assessment and associated plans will provide the basis for a common understanding of stakeholders of the risks for that trial, and facilitate a risk-proportionate approach to trial activities.

The guidance is available on the MHRA and NETSCC websites.

